# Supplementary material for: Heat shock protein gp96 drives natural killer cell maturation and anti-tumor immunity by counteracting Trim28 to stabilize Eomes
Source: Nat Commun. 2024 Feb 6;15:1106. doi: 10.1038/s41467-024-45426-5 (PMC10847424; doi:10.1038/s41467-024-45426-5)
Supplement: Supplementary file 1 — Supplementary Information [file 41467_2024_45426_MOESM1_ESM.pdf]

Supplementary information to:

Heat shock protein gp96 drives natural killer cell maturation and anti-tumor immunity by counteracting Trim28 to stabilize Eomes

Yuxiu Xu[1, #], Xin Li[1, #, *] and Songdong Meng[1, 2*]

`

This PDF file includes:

- **Supplemental Figures S1-S8**
- **Supplemental Tables S1-S4**

Supplementary Figures

Supplementary Figure 1

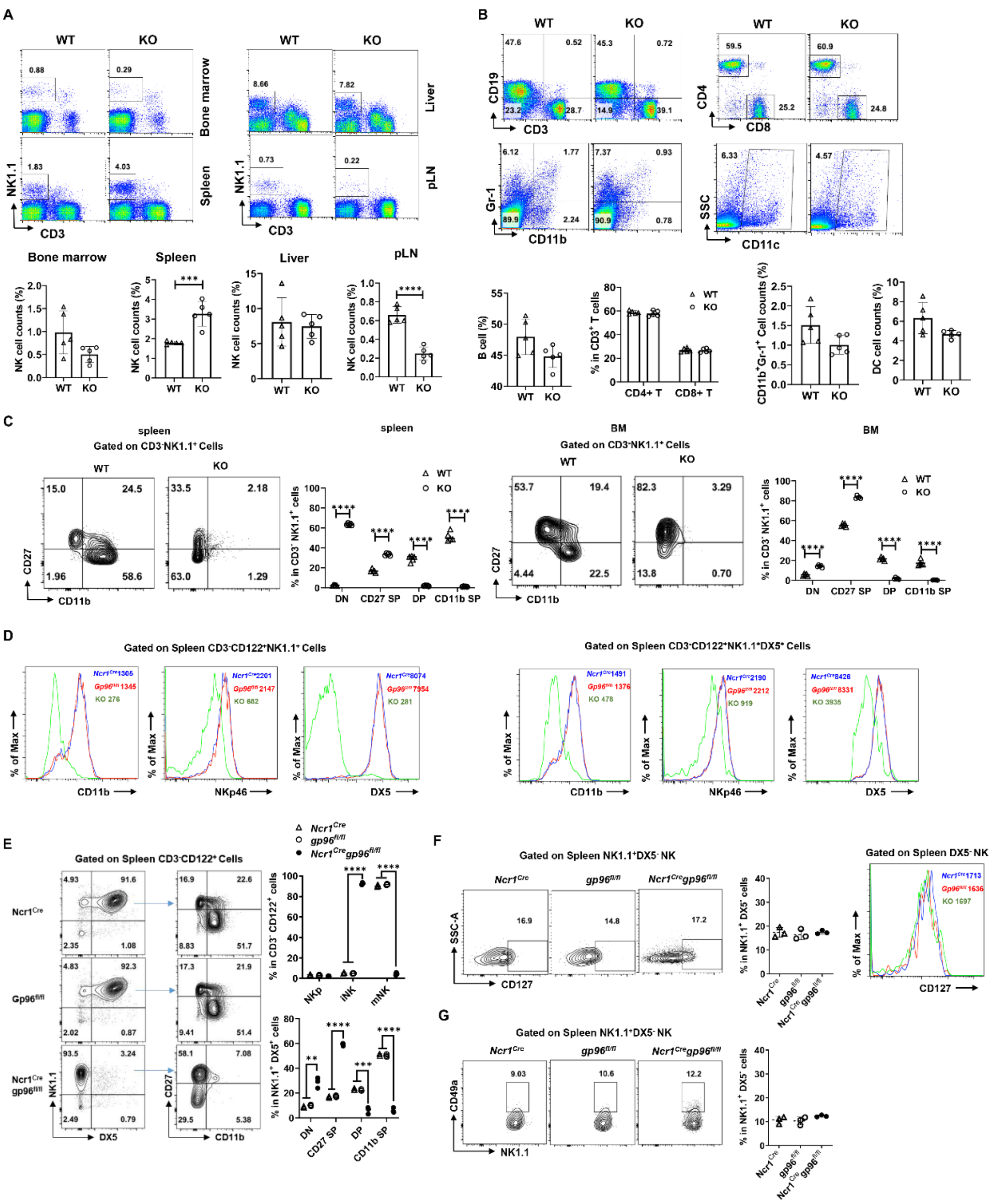

Supplementary Figure 1. NK-specific gp96 deficiency reduces NK cell maturation. (A) Frequency and enumeration of NK cells in the indicated organs (pLN, periphery lymph node). (B) Flow cytometry analysis of B cell, T cell, MDSC and DC cell in the spleen of WT and KO mice. (C) Representative flow cytometry plots showing the percentages of CD27$^-$CD11b$^-$ (DN), CD27$^+$CD11b$^-$ (CD27 SP), CD27$^+$CD11b$^+$ (DP), and CD27$^-$CD11b$^+$ (CD11b SP) cells on gated CD3$^-$NK1.1$^+$ splenocytes and bone

marrow cells from WT and gp96-deficient mice. (D-E) Representative flow cytometry analysis of indicated marker levels (D) and percentages of different subsets (E) in spleen of $Ncr1^{Cre}$ mice, $gp96^{fl/fl}$ and $Ncr1^{Cre}gp96^{fl/fl}$ mice. (F-G) Representative flow cytometry analysis of CD127 (F) and CD49a (G) levels in spleen of $Ncr1^{Cre}$ mice, $gp96^{fl/fl}$ and $Ncr1^{Cre}gp96^{fl/fl}$ mice. Mean ± SD is shown. The data are representative of two independent experiments with similar results. The data are representative of two independent experiments with similar results. Dots represent data from n = 5 mice/group (A-C) and n = 3 mice/group (E-G) Statistical significance was determined using two-tailed unpaired t test. ***p*< 0.01, ****p*< 0.001, *****p*< 0.0001. *p* values: (A) *p*=0.0008 (spleen), *p*<0.0001 (pLN), (C) *p*<0.0001 (spleen, DN), *p*<0.0001 (spleen, CD27 SP), *p*<0.0001 (spleen, DP), *p*<0.0001 (spleen, CD11b SP), *p*<0.0001 (BM, DN), *p*<0.0001 (BM, CD27 SP), *p*<0.0001 (BM, DP), *p*<0.0001 (BM, CD11b SP), (E) *p*<0.0001 (iNK), *p*<0.0001 (mNK), *p*=0.0013 (DN, $Ncr1^{Cre}gp96^{fl/fl}$ vs $Ncr1^{Cre}$), *p*=0.0016 (DN, $Ncr1^{Cre}gp96^{fl/fl}$ vs $gp96^{fl/fl}$), *p*<0.0001 (CD27 SP, $Ncr1^{Cre}gp96^{fl/fl}$ vs $Ncr1^{Cre}$), *p*<0.0001 (CD27 SP, $Ncr1^{Cre}gp96^{fl/fl}$ vs $gp96^{fl/fl}$), *p*=0.0005 (DP, $Ncr1^{Cre}gp96^{fl/fl}$ vs $Ncr1^{Cre}$), *p*=0.0005 (DP, $Ncr1^{Cre}gp96^{fl/fl}$ vs $gp96^{fl/fl}$), *p*<0.0001 (CD11b SP, $Ncr1^{Cre}gp96^{fl/fl}$ vs $Ncr1^{Cre}$), *p*<0.0001 (CD11b SP, $Ncr1^{Cre}gp96^{fl/fl}$ vs $gp96^{fl/fl}$).

Supplementary Figure 2

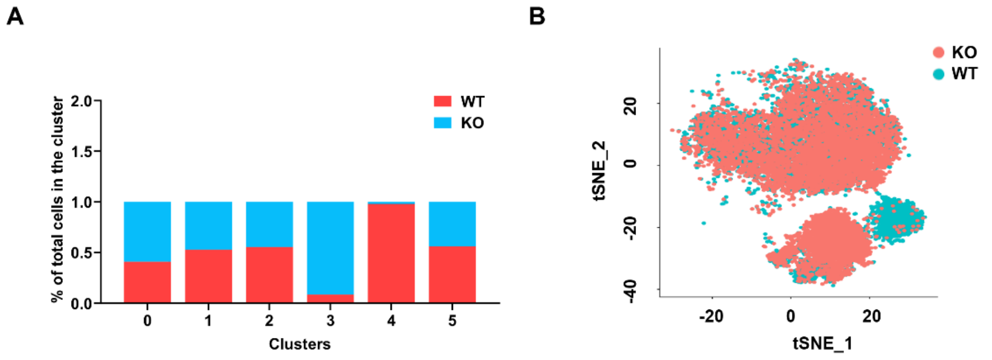

Supplementary Figure 2. Analysis of high-throughput single-cell RNA-seq of NK cells from WT and KO mice. (A) Percentages of WT and gp96 KO NK cells within each cluster were calculated. (B) t-Distributed Stochastic Neighbor Embedding (tSNE) and graph visualization of NK cells from two analyzed mouse strains.

Supplementary Figure 3

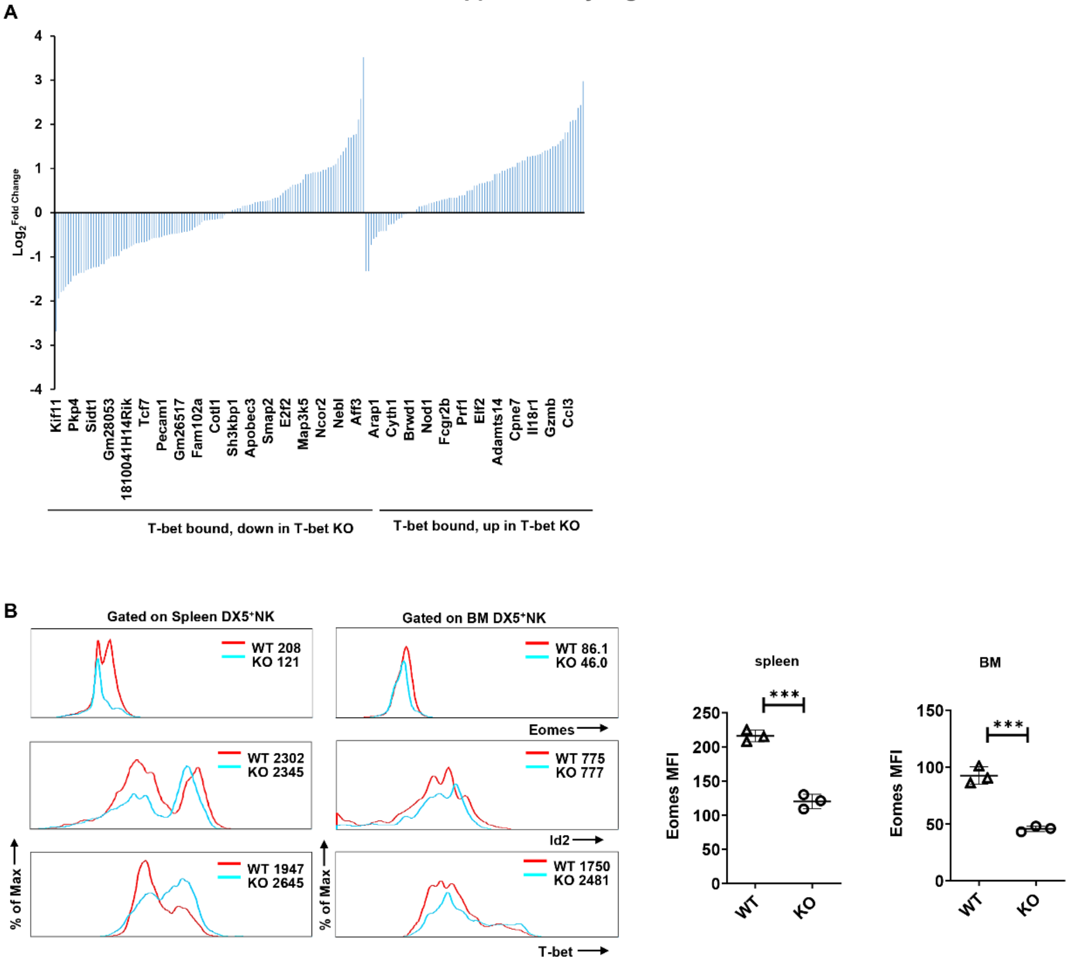

Supplementary Figure 3. Effect of gp96 on transcription factor expression in NK cells. (A) Correlation analysis of expression of indicated T-bet-bound genes in DX5^{+} splenic NK cells was analyzed. The fold change indicates the difference in relative transcript expression between WT compared with *Ncr1*^{Cre}*gp96*$^{\mathrm{fl/fl}}$ mice, as determined by RNA-seq. (B) Flow cytometry analysis of levels of indicated transcription factors between *Ncr1*^{Cre}*gp96*$^{\mathrm{fl/fl}}$ and WT NK cells. The data are representative of two independent experiments with similar results. n = 3 mice/group. Mean ± SD is shown. Statistical significance was determined using two-tailed unpaired t test. ****p*< 0.001. *p* values: (B) *p*=0.0003 (spleen), *p*=0.0008 (BM).

Supplementary Figure 4

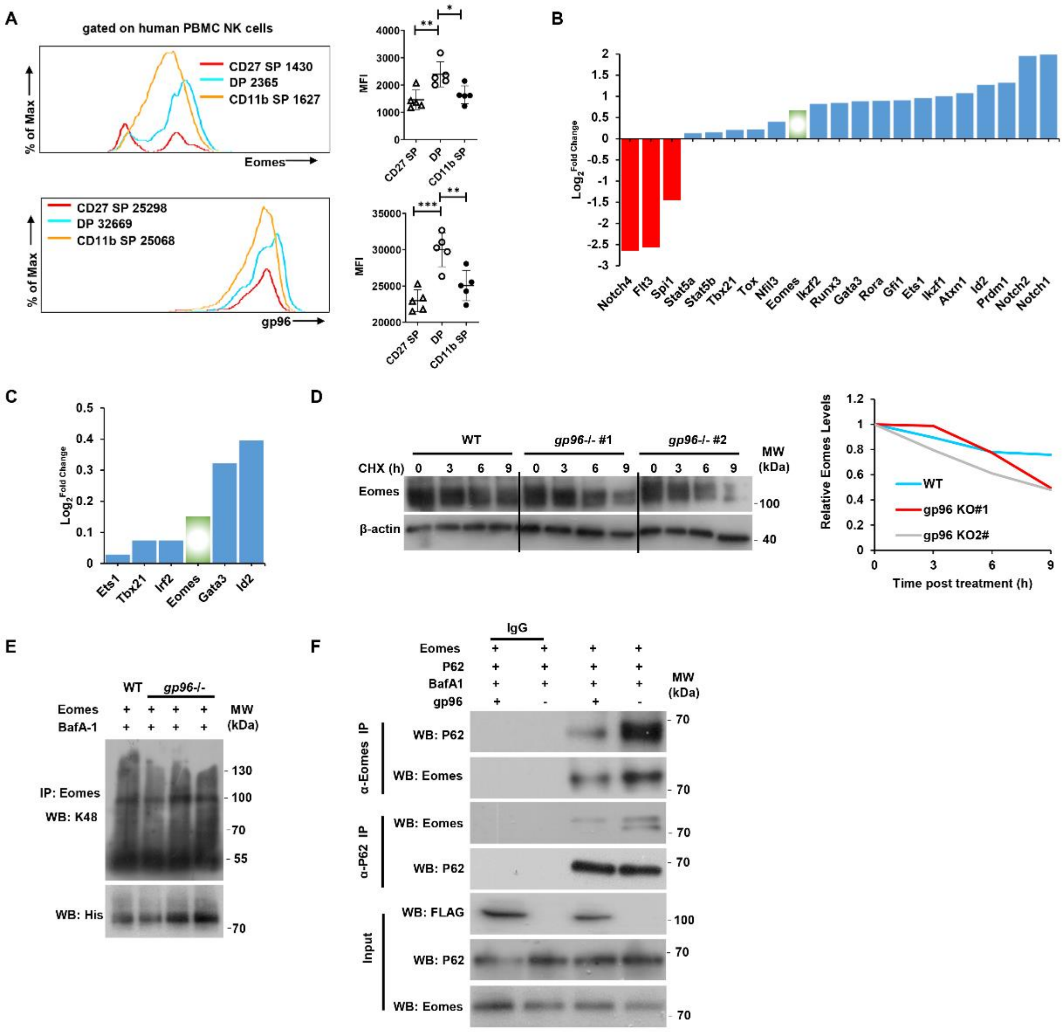

Supplementary Figure 4. Gp96 regulates Eomes expression at the posttranscriptional level. (A) Flow cytometry analysis of Eomes and gp96 levels among CD27 single positive, double positive (DP), CD11b single positive NK cells in human PBMC. n = 5 samples/group. Mean ± SD is shown. Statistical significance was determined using two-tailed unpaired t test. *$p < 0.05$, **$p < 0.01$, ***$p < 0.001$. p values: p=0.0071 (Eomes, DP vs CD27 SP), p=0.0179 (Eomes, CD11b SP vs DP), p=0.0005 (gp96, DP vs CD27 SP), p=0.0077 (gp96, CD11b SP vs DP). (B) Expression of indicated TF (transcription factors) as determined by RNA-seq. The fold change indicates the difference in relative transcript expression between WT compared with *Ncr1*^{Cre}*gp96*$^{\text{fl/fl}}$ mice. (C) Splenic NK cells were sorted from *Ncr1*^{Cre}*gp96*$^{\text{fl/fl}}$ and WT mice. Cells were lysed for RNA extraction. The expression of transcription factor mRNA was analyzed using qPCR. The fold change indicates the difference in relative

transcript expression between WT compared with *Ncr1*^{Cre}*gp96*$^{\mathrm{fl/fl}}$ mice. (D) Western blot analysis of Eomes levels in WT and gp96 KO HEK293 cells. Cells were transfected with His-Eomes before being treated with 50 μg/ml CHX for the time indicated. (E) WT and gp96 knockout HEK293 cells were transfected with His-Eomes. Cells were grown for 24 h and treated with 20 nM of BafA1 for 4 h, followed by IP-Western analyses. (F) HEK293 cells were co-transfected with His-Eomes and indicated plasmids. Cells were then treated as (Fig. 7I) and subjected to IP-Western analyses. The data are representative of two independent experiments with similar results.

Supplementary Figure 5

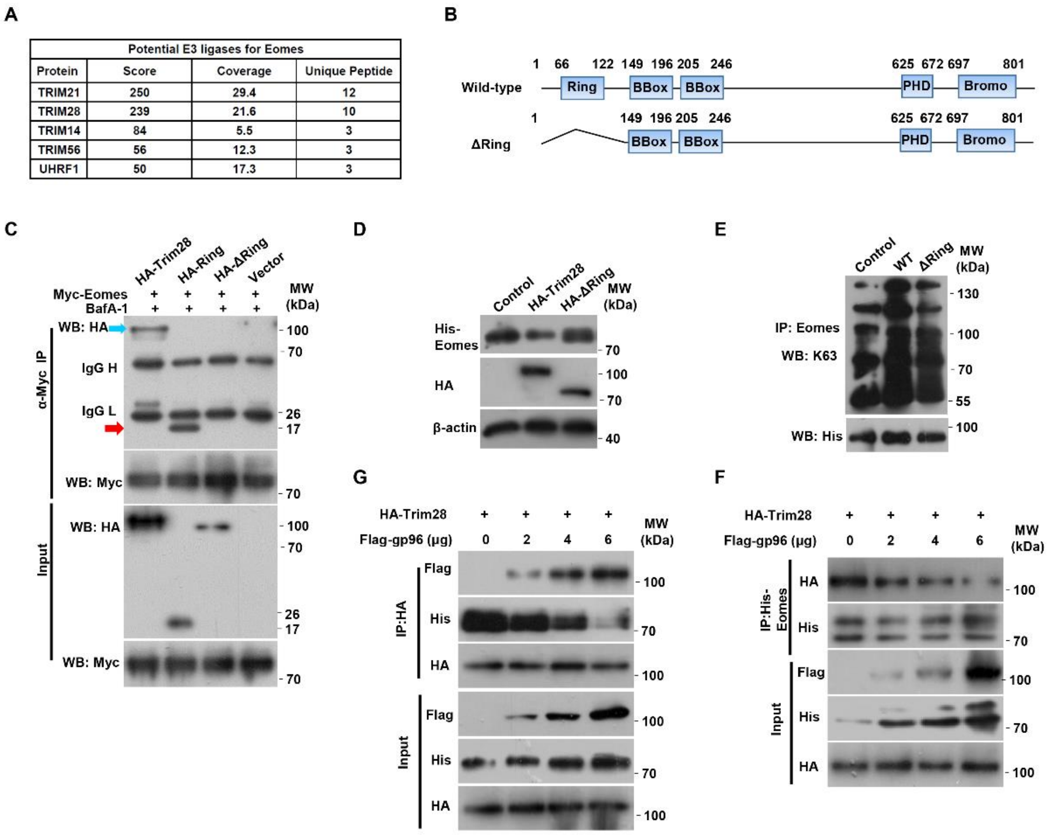

A

Potential E3 ligases for Eomes			
Protein	Score	Coverage	Unique Peptide
TRIM21	250	29.4	12
TRIM28	239	21.6	10
TRIM14	84	5.5	3
TRIM56	56	12.3	3
UHRF1	50	17.3	3

Supplementary Figure 5. Trim28 mediates ubiquitination of Eomes through RING domain. (A) Mouse splenic NK cells were sorted and treated with 20 nM of BafA1 for 4 h, followed by immunoprecipitation with Eomes antibody. The sample was loaded for SDS-PAGE and Mass Spectrometry. Potential E3-ubiquitin ligases were listed. (B) A schematic representation of Trim28 deletion mutants and fragments was used in this study. (C) HEK293 cells stably expressing Eomes were transfected with indicated plasmids. Cells were grown for 24 h and treated with 20 nM of BafA1 for 4 h, followed by IP-Western analyses. Red arrow represents RING-Trim28; Blue arrow represents full-length Trim28. (D and E) HEK293 cells stably expressing Eomes were transfected with indicated plasmids. Cells were grown for 24 h before being subjected to Western blot (D) and IP-Western (E) analyses. (F and G) HEK293 cells stably expressing Eomes were transfected with HA-Trim28 and Flag-gp96 plasmid at the indicated amounts. Cells were grown for 24 h, followed by IP-Western analyses. The data are representative of two independent experiments with similar results.

Supplementary Figure 6

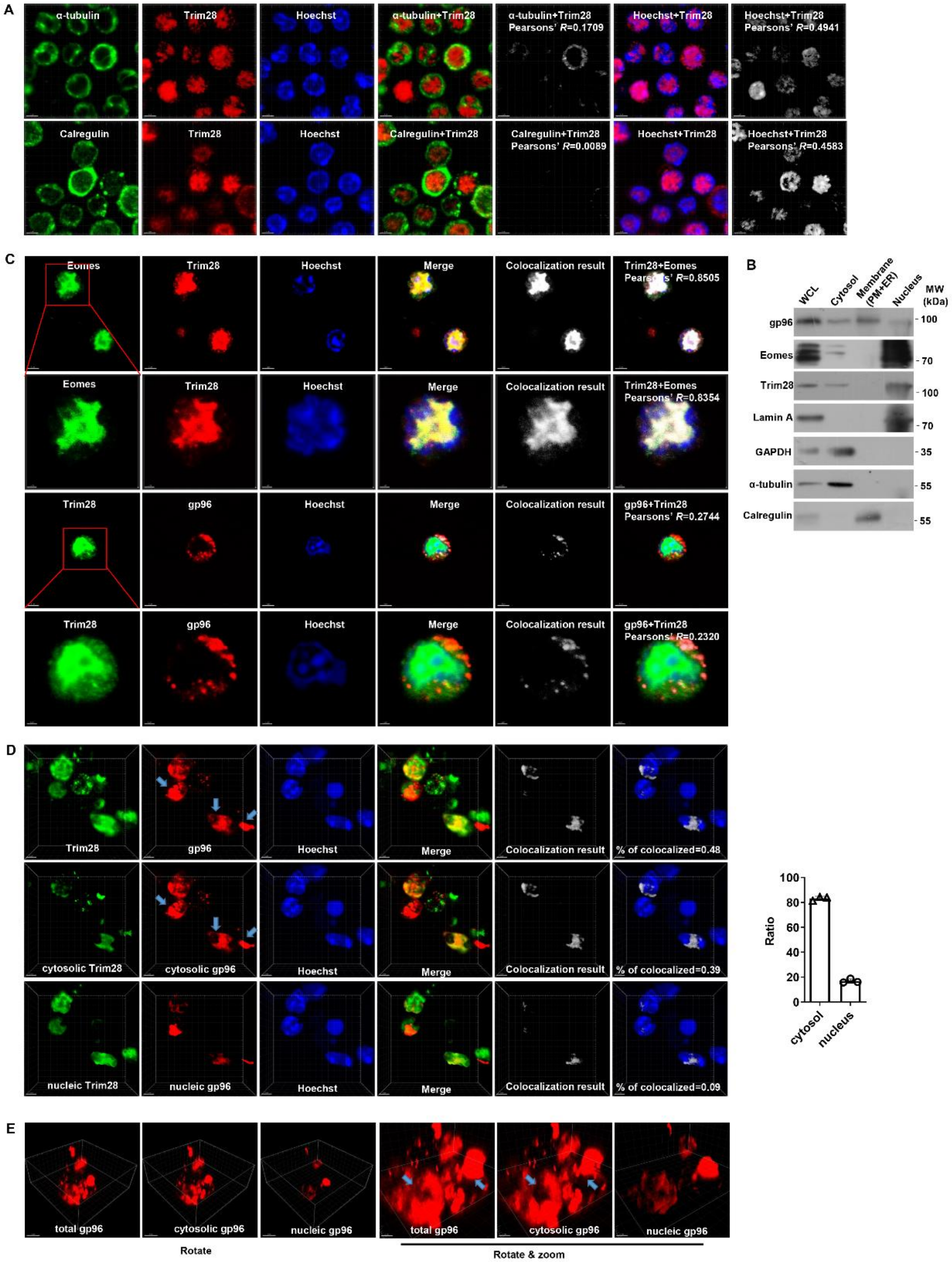

Supplementary Figure 6

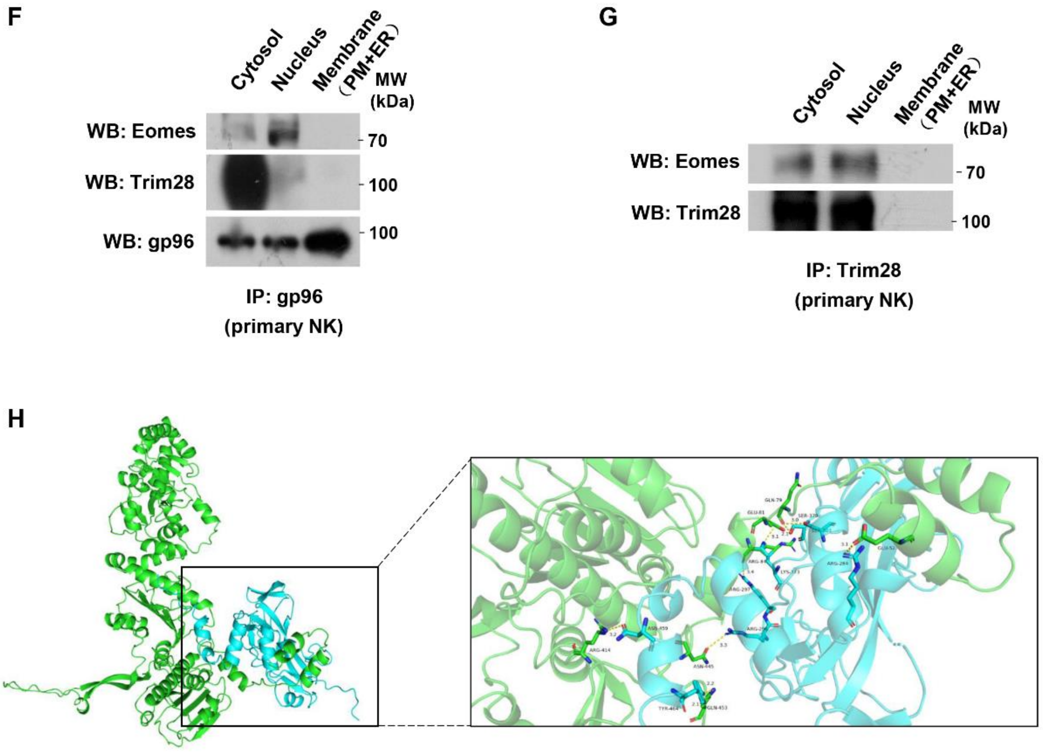

Supplementary Figure 6. The interaction sites among gp96, Eomes, and Trim28. (A) Super-resolution immunofluorescence imaging (SIM imaging) of CD11b^{+}CD27^{+} NK cells stained with Trim28 and the ER marker calregulin, cytosolic marker tubulin, or Hoechst 33342 for cell nuclei staining. Scale bars, 3 μm. (B) Representative western blotting of gp96, Eomes and Trim28 isolated from distinct cell extracts. Whole cell lysate (WCL) served as a positive control. Calregulin was served as ER marker, and α-Tubulin and GAPDH were served as cytosol markers, and lamin was served as nucleic marker. (C) SIM imaging of CD11b^{+}CD27^{+} NK cells stained with Eomes, gp96 and Trim28. Scale bars, 3 μm for original shots and 1 μm for magnified views. (D) Z-stack images of gp96 and Trim28 in whole cell, cytosol and nucleus, respectively. Percentage of ROI colocalized for cytosol and nucleus were obtained by Imaris. Percentages of colocalization of gp96 and Trim28 in the nucleus and cytosol were calculated, respectively. Scale bars, 3 μm. Arrows show the differences between total and cytosolic gp96 images. Dots represent data from n=3 fields. (E) 3D images of gp96 in whole cell, cytosol and nucleus, respectively. Scale bars, 5 μm and 2 μm,

respectively. Arrows indicate the differences between total and cytosolic gp96 images. (F-G) Cell fractions were harvested in primary NK cells and then subjected to IP-Western analyses. (H) The protein-protein interaction prediction tools-ZDOCK 3.0.2 were used to predict the interaction between Eomes (PDB ID: AF-O54839) and gp96 (PDB ID: AF-P14625). Blue represents Eomes, and the green represents gp96. Data are representative of three independent experiments with similar results.

Supplementary Figure 7

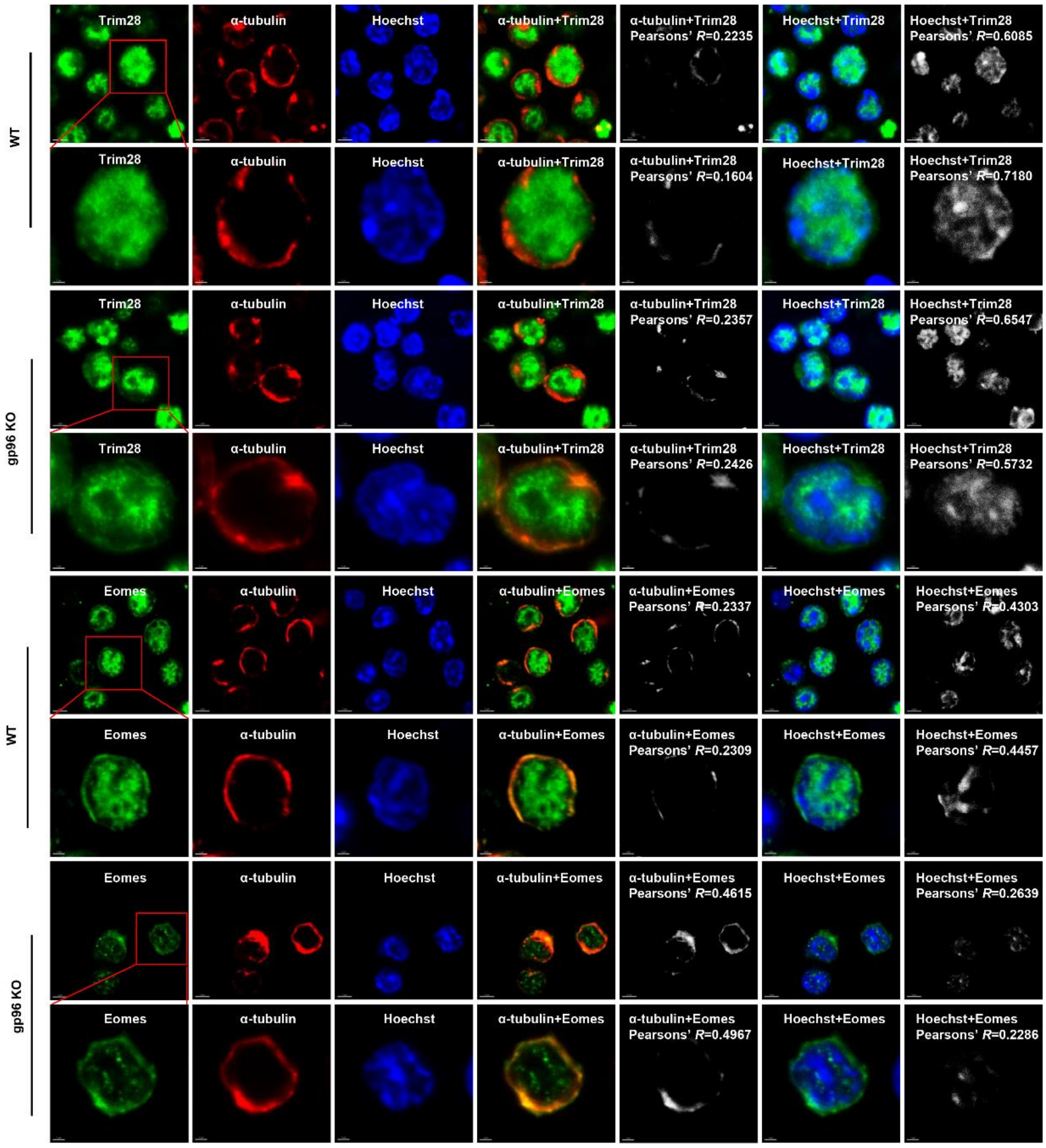

Supplementary Figure 7. SIM imaging of NK cells from WT and gp96 KO mice stained with Eomes or Trim28. α-Tubulin was served as cytosol marker. Scale bars, 3 μm for original shots and 1 μm for magnified views. Data are representative of three independent experiments with similar results.

Supplementary Figure 8

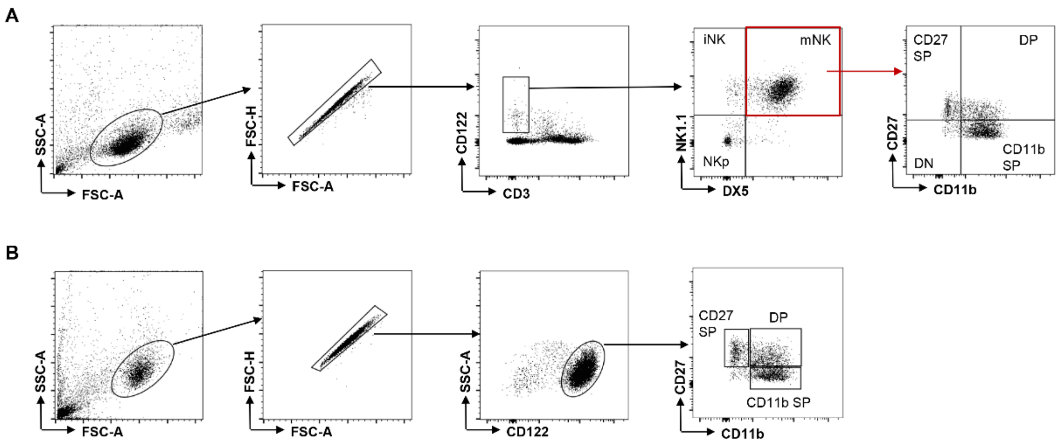

Supplementary Figure 8. Gating/sorting strategies used in this study. (A) Flow cytometry gating strategies for flow cytometry analysis presented on Fig.1. (B) Gating strategy to sort $CD11b^{-}CD27^{+}$, $CD11b^{+}CD27^{+}$, and $CD11b^{+}CD27^{-}$ NK cells from WT mice for immunostaining analysis presented on Fig.6B-E, Fig. S6A, S6C-E and Fig.S7.

Supplementary Tables

Table S1. Primers for genotyping the mice.

Primer	Sequence
floxed *gp96* forward(5'-3')	TGCCAGAGACTACAATTCCCAGCA
floxed *gp96* reverse(5'-3')	AAACACGAACTCACCAATCGTGCC
Ncr1 cre-WT-forward(5'-3')	CGAATTGGTCTGGCATGCATAATC
Ncr1 cre-WT-reverse(5'-3')	CTTTGCCAAACTTGGTAACACTCC
Ncr1 cre-Mut-reverse(5'-3')	CACACCGGCCTTATTCCAAG

Table S2. Primers for qRT-PCR.

Primer	Sequence (5'-3')
Ets-1-F	CCCTGGGTAAAGAATGCTTCC
Ets-1-R	GCTGATGAAGTAATCCGAGGTG
Tbx21-F	AGCAAGGACGGCGAATGTT
Tbx21-R	GTGGACATATAAGCGGTTCCC
Irf2-F	AATTCCAATACGATACCAGGGCT
Irf2-R	GAGCGGAGCATCCTTTTCCA
Eomes-F	GGCCCCTATGGCTCAAATTCC
Eomes-R	GAACCACTTCCACGAAAACATTG
Gata3-F	AAGCTCAGTATCCGCTGACG
Gata3-R	GTTTCCGTAGTAGGACGGGAC
Id2-F	ATGAAAGCCTTCAGTCCGGTG
Id2-R	AGCAGACTCATCGGGTCGT
Gapdh-F	TGACCACAGTCCATGCCATC
Gapdh-R	GATGGGGGTTACACAGGCAG

Table S3. Antibodies for immunoblotting and immunofluorescence.

Target antigen	Vendor or Source	Catolog#	Working concentration
GAPDH	Cell Signaling Technology	5174	WB 1:1000
β-actin	Cell Signaling Technology	3700	WB 1:1000
phospho-S6	Cell Signaling Technology	4858	WB 1:1000
phospho-Stat5 (Tyr694)	Cell Signaling Technology	4322	WB 1:1000
gp96	Cell Signaling Technology	20292	WB 1:1000<br>IF 1:100
Trim28	Cell Signaling Technology	4124	WB 1:1000<br>IF 1:100
His-Tag	Cell Signaling Technology	12698	WB 1:1000
α-Tubulin	Cell Signaling Technology	3873	WB 1:1000<br>IF 1:100
Calregulin	Santa Cruz Biotechnology	sc-166837	WB 1:1000<br>IF 1:100
gp96	Santa Cruz Biotechnology	sc-393402	WB 1:1000<br>IF 1:100
Eomes	eBioscience	14-4875-82	WB 1:1000
Eomes	Abcam	Ab216870	IF 1:100
Myc-tag	Bioworld	AP0031M	WB 1:1000
DDDDK-tag	Bioworld	AP0007M	WB 1:1000
GFP-tag	Bioworld	AP0675M	WB 1:1000
HA-tag	Bioworld	AP0005M	WB 1:1000
Alexa-Fluor 488 Goat Anti-Rabbit lgG	Cell Signaling Technology	4412	IF 1:200
Alexa-Fluor 488 Goat Anti-Mouse lgG	Cell Signaling Technology	4408	IF 1:200

Alexa-Fluor 594 Goat Anti-Rabbit lgG	Cell Signaling Technology	8889	IF 1:200
Alexa-Fluor 594 Goat Anti-Mouse lgG	Cell Signaling Technology	8890	IF 1:200
HRP-Goat Anti-Mouse lgG	Cell Signaling Technology	7076	WB 1:2000
HRP-Goat Anti-Rabbit lgG	Cell Signaling Technology	7074	WB 1:2000
HRP-Goat Anti-Rat lgG	Cell Signaling Technology	7077	WB 1:2000

Table S4. Antibodies for flow cytometry.

Target antigen	Vendor or Source	Catolog#	Working concentration
anti-mouse CD3	BioLegend	100214	1:500
anti-mouse CD49b	BioLegend	108908	1:500
anti-mouse NK1.1	BioLegend	108713	1:500
anti-mouse/human CD11b	BioLegend	101263	1:500
anti-mouse CD45	BioLegend	103108	1:500
anti-mouse CD11c	BioLegend	117333	1:500
anti-mouse F4/80	BioLegend	123149	1:500
anti-mouse CD27	BioLegend	124229	1:500
anti-mouse KLRG1	BioLegend	138410	1:500
anti-mouse CD122	BioLegend	123219	1:500
anti-mouse NKp46	BioLegend	137611	1:500
anti-mouse IFN-γ	BioLegend	505832	1:500
anti-mouse CD19	BioLegend	115507	1:500
anti-mouse CD4	BioLegend	100433	1:500
anti-mouse CD49a	BioLegend	142605	1:500
anti-mouse CD127	BioLegend	135307	1:500
anti-mouse CD107a	BioLegend	121611	1:500
anti-human CD27	BioLegend	302824	1:500
anti-Eomes	Thermo Fisher Scientific	53-4875-82	1:500
anti-Id2	Thermo Fisher Scientific	17-9475-82	1:500
anti-T-bet	Thermo Fisher Scientific	12-5825-80	1:500
anti-Foxp3	Thermo Fisher Scientific	17-5773-82	1:500
anti-CD8a	Thermo Fisher Scientific	45-0081-80	1:500
anti-human EOMES	Thermo Fisher Scientific	12-4877-42	1:500
anti-human CD3	Thermo Fisher Scientific	11-0038-80	1:500

anti-human CD56	Thermo Fisher Scientific	17-0567-42	1:500
anti-human CD45	Thermo Fisher Scientific	45-0459-41	1:500
PE-conjugated grp94 monoclonal antibody	Enzo Life Sciences	ADI-SPA-850PE	1:500
