# Supplementary material for: Heat shock protein gp96 drives natural killer cell maturation and anti-tumor immunity by counteracting Trim28 to stabilize Eomes
Source: Nat Commun. 2024 Feb 6;15:1106. doi: 10.1038/s41467-024-45426-5 (PMC10847424; doi:10.1038/s41467-024-45426-5)
Supplement: Supplementary file 3 — Description of Additional Supplementary Files [file 41467_2024_45426_MOESM3_ESM.pdf]

DESCRIPTION OF ADDITIONAL SUPPLEMENTARY FILES DOCUMENT

Supplementary Movie 1. 3D video of localization of gp96 and Eomes in cytosol.

Supplementary Movie 2. 3D video of localization of gp96 and Trim28 in cytosol.

Supplementary Movie 3. 3D video of localization of gp96 and Eomes in nucleus.

Supplementary Movie 4. 3D video of localization of gp96 and Trim28 in nucleus
