# Supplementary material for: Heat shock protein gp96 drives natural killer cell maturation and anti-tumor immunity by counteracting Trim28 to stabilize Eomes
Source: Nat Commun. 2024 Feb 6;15:1106. doi: 10.1038/s41467-024-45426-5 (PMC10847424; doi:10.1038/s41467-024-45426-5)
Supplement: Supplementary file 8 — Reporting Summary [file 41467_2024_45426_MOESM8_ESM.pdf]

nature portfolio

Corresponding author(s): Songdong Meng

Last updated by author(s): Jan 7, 2024

Reporting Summary

Nature Portfolio wishes to improve the reproducibility of the work that we publish. This form provides structure for consistency and transparency in reporting. For further information on Nature Portfolio policies, see our Editorial Policies and the Editorial Policy Checklist.

Statistics

For all statistical analyses, confirm that the following items are present in the figure legend, table legend, main text, or Methods section.

n/a	Confirmed	
☐	☒	The exact sample size (*n*) for each experimental group/condition, given as a discrete number and unit of measurement
☐	☒	A statement on whether measurements were taken from distinct samples or whether the same sample was measured repeatedly
☐	☒	The statistical test(s) used AND whether they are one- or two-sided<br>*Only common tests should be described solely by name; describe more complex techniques in the Methods section.*
☐	☒	A description of all covariates tested
☐	☒	A description of any assumptions or corrections, such as tests of normality and adjustment for multiple comparisons
☐	☒	A full description of the statistical parameters including central tendency (e.g. means) or other basic estimates (e.g. regression coefficient) AND variation (e.g. standard deviation) or associated estimates of uncertainty (e.g. confidence intervals)
☐	☒	For null hypothesis testing, the test statistic (e.g. *F*, *t*, *r*) with confidence intervals, effect sizes, degrees of freedom and *P* value noted<br>*Give P values as exact values whenever suitable.*
☒	☐	For Bayesian analysis, information on the choice of priors and Markov chain Monte Carlo settings
☒	☐	For hierarchical and complex designs, identification of the appropriate level for tests and full reporting of outcomes
☐	☒	Estimates of effect sizes (e.g. Cohen's *d*, Pearson's *r*), indicating how they were calculated

Our web collection on statistics for biologists contains articles on many of the points above.

Software and code

Policy information about availability of computer code

Data collection	Splenic NK cells pooled from three mice of each indicated genotyped were enriched by FACS for library preparation, and scRNA-seq was performed. The final library pool was sequenced on the Illumina NovaSeq 6000 instrument using 150-base pair paired-end reads. Raw sequencing data were converted to FASTQ files and aligned to the mouse genome reference sequence (GRCH38). For sc-RNA seqeucing, we used CellRanger software (version 3.0.1) to generate the single cell information.
Data analysis	For sc-RNA-sequencing, the 10X Genomics Cell Ranger (version 3.0.1) was used to demultiplex samples, process barcodes, and generate a digital gene-cell matrix from this data. We excluded doublets, poor-quality cells and contamination cells by Seurat software (version 3.1.5). After quality control and filtering steps, 7349 cells from Ncr1Cre mice and 8350 cells from Ncr1Cregp96fl/fl mice were used for further analyses. The Louvain algorithm was used for the unsupervised computational analysis of scRNA-seq data.<br>For flow cytometry data analysis, we used FlowJo (version 10) and numerical data were further analyzed by using GraphPad Prism (version 6) for statistical analysis. For confocol analysis, we used Imaris (version 9.7.2).

For manuscripts utilizing custom algorithms or software that are central to the research but not yet described in published literature, software must be made available to editors and reviewers. We strongly encourage code deposition in a community repository (e.g. GitHub). See the Nature Portfolio guidelines for submitting code & software for further information.

Data

Policy information about availability of data

All manuscripts must include a data availability statement. This statement should provide the following information, where applicable:

- Accession codes, unique identifiers, or web links for publicly available datasets
- A description of any restrictions on data availability
- For clinical datasets or third party data, please ensure that the statement adheres to our policy

The RNA-seq data generated in this study have been deposited in the National Center for Biotechnology Information Sequence Read Archive (NCBI SRA) under accession number PRJNA870662. http://www.ncbi.nlm.nih.gov/bioproject/PRJNA870662.
The scRNA seq data generated in this study have been deposited in the National Center for Biotechnology Information Sequence Read Archive (NCBI SRA) under accession number PRJNA872215. http://www.ncbi.nlm.nih.gov/bioproject/PRJNA872215.

Research involving human participants, their data, or biological material

Policy information about studies with human participants or human data. See also policy information about sex, gender (identity/presentation), and sexual orientation and race, ethnicity and racism.

Reporting on sex and gender	Blood samples were collected from 5 healthy volunteers, including 3 women and 2 men. Informed consent from all participants has been obtained. Sex and gender were not considered in this study.
Reporting on race, ethnicity, or other socially relevant groupings	All the volunteers are Chinese. They were not categorized.
Population characteristics	See above.
Recruitment	All participants were recruited in our lab.
Ethics oversight	All experiments were approved by the Fifth Medical Center of PLA General Hospitol (permit number KY-2020-11-5-1.)

Note that full information on the approval of the study protocol must also be provided in the manuscript.

Field-specific reporting

Please select the one below that is the best fit for your research. If you are not sure, read the appropriate sections before making your selection.

☒ Life sciences ☐ Behavioural & social sciences ☐ Ecological, evolutionary & environmental sciences

For a reference copy of the document with all sections, see nature.com/documents/nr-reporting-summary-flat.pdf

Life sciences study design

All studies must disclose on these points even when the disclosure is negative.

Sample size	Our study was not a population study, and sample size was not predetermined. For animal studies, a minimum sample size of 3 animals for control and experimental groups was used in each assay. These numbers were are determined by previous experiments in our laboratory in order to achieve 95% power for two-sided t-test. For human studies, sample size was determined by the number of cells.
Data exclusions	No data were excluded from the analyses.
Replication	As stated in the figure legends, the data are representative of two or three independent experiments with similar results.
Randomization	For animal studies, assignment to groups was by mouse genotype (e.g. wildtype or knockout). Experiments involving human samples do not need to be categorized.
Blinding	No blinding was performed. The samples were prepared, treated and analyzed by the same standard procedure. The investgators did not expect the experiment results and most of the test data was automatically generated by the instrument.

Behavioural & social sciences study design

All studies must disclose on these points even when the disclosure is negative.

Study description	

Research sample

Sampling strategy

Data collection

Timing

Data exclusions

Non-participation

Randomization

Ecological, evolutionary & environmental sciences study design

All studies must disclose on these points even when the disclosure is negative.

Study description

Research sample

Sampling strategy

Data collection

Timing and spatial scale

Data exclusions

Reproducibility

Randomization

Blinding

Did the study involve field work? ☐ Yes ☐ No

Field work, collection and transport

Field conditions

Location

Access & import/export

Disturbance

Reporting for specific materials, systems and methods

We require information from authors about some types of materials, experimental systems and methods used in many studies. Here, indicate whether each material, system or method listed is relevant to your study. If you are not sure if a list item applies to your research, read the appropriate section before selecting a response.

Materials & experimental systems

n/a	Involved in the study
☐	☒ Antibodies
☐	☒ Eukaryotic cell lines
☒	☐ Palaeontology and archaeology
☐	☒ Animals and other organisms
☒	☐ Clinical data
☒	☐ Dual use research of concern
☒	☐ Plants

Methods

n/a	Involved in the study
☒	☐ ChIP-seq
☐	☒ Flow cytometry
☒	☐ MRI-based neuroimaging

Antibodies

Antibodies used

Primary antibodies used include:
anti-GAPDH (Cell Signaling Technology 5174, 1:1000 for WB).
anti-β-actin (Cell Signaling Technology 3700, 1:1000 for WB).
anti-phospho-S6 (Cell Signaling Technology 4858, 1:1000 for WB).
anti-phosph-Stat5(Tyr694)(Cell Signaling Technology 4322, 1:1000 for WB).
anti-gp96 (Cell Signaling Technology 20292, 1:1000 for WB, 1:100 for IF).
anti-Trim28 (Cell Signaling Technology 4124, 1:1000 for WB, 1:100 for IF).
anti-His-Tag (Cell Signaling Technology 12698, 1:1000 for WB).
anti-α-Tubulin (Cell Signaling Technology 3873, 1:1000 for WB, 1:100 for IF).
anti-Calreguln(Santa Cruz Biotechnology sc-166837,1:1000 for WB,1:100 for IF).
anti-gp96(Santa Cruz Biotechnology sc-393402,1:1000 for WB,1:100 for IF).
anti-Eomes(eBioscience 14-4875-82,1:1000 for WB).
anti-Eomes(Abacm Ab216870,1:100 for IF).
anti-Myc-tag(Bioword AP0031M,1:1000 for WB).
anti-DDDDK-tag(Bioworld AP0007M,1:1000 for WB).
anti-GFP-tag(Bioworld AP0675M,1:1000 for WB).
anti-HA-tag(Bioworld AP0005M,1:1000 for WB).
Secondary antibodies used include:
Alexa-Fluor 488 Goat Anti-Rabbit lgG(Cell Signaling Technology 4412,1:200 for IF).
Alexa-Fluor 488 Goat Anti-Mouse lgG(Cell Signaling Technology 4408,1:200 for IF).
Alexa-Fluor 594 Goat Anti-Rabbit lgG(Cell Signaling Technology 8889,1:200 for IF).
Alexa-Fluor 594 Goat Anti-Mouse lgG(Cell Signaling Technology 8890,1:200 for IF).
HRP-Goat Anti-Mouse lgG(Cell Signaling Technology 7076,1:2000 for WB).
HRP-Goat Anti-Rabbit lgG(Cell Signaling Technology 7074,1:2000 for WB).
HRP-Goat Anti-Rat lgG(Cell Signaling Technology 7077,1:2000 for WB).
Antibodies for flow cytometry:
anti-mouse CD3(BioLegent 100214,1:500 for FC).
anti-mouse CD49b(BioLegent 108908,1:500 for FC).
anti-mouse NK1.1(BioLegent 108713,1:500 for FC).
antii-mouse/human CD11b(BioLegent 101263,1:500 for FC).
anti-mouse CD45(BioLegent 103108,1:500 for FC).
anti-mouse CD11c(BioLegent 117333,1:500 for FC).
anti-mouse F4/80(BioLegent 123149,1:500 for FC).
anti-mouse CD27(BioLegent 124229,1:500 for FC).
anti-mouse KLRG1(Biolegent 138410,1:500 for FC).
anti-mouse CD112(Biolegent 123219,1:500 for FC).
anti-mouse NKp46(Biolegent 137611,1:500 for FC).
anti-mouse IFN-γ(Biolegent 505832,1:500 for FC).
anti-mouse CD19(BioLegent 115507,1:500 for FC).
anti-mouse CD4(BioLegent 100433,1:500 for FC).
anti-mouse CD49a(BioLegent 142605,1:500 for FC).
anti-mouse CD127(BioLegent 135307,1:500 for FC).
anti-mouse CD107a(BioLegent 121611,1:500 for FC).
anti-human CD27(BioLegent 302824,1:500 for FC).
anti-Eomes(Thermo Fisher Scientific 53-4875-82,1:500 for FC).
anti-Id2(Thermo Fisher Scientific 17-9475-82,1:500 for FC).
anti-T-bet(Thermo Fisher Scientific 12-5825-80,1:500 for FC).
anti-Foxp3(Thermo Fisher Scientific 17-5773-82,1:500 for FC).
anti-CD8a(Thermo Fisher Scientific 45-0081-80,1:500 for FC).
anti-human EOMES(Thermo Fisher Scientific 12-4877-42,1:500 for FC).
anti-human CD3(Thermo Fisher Scientific 11-0038-80,1:500 for FC).
anti-human CD56(Thermo Fisher Scientific 17-0567-42,1:500 for FC).
anti-human CD45(Thermo Fisher Scientific 45-0459-41,1:500 for FC).
PE-conjugated grp94 monoclonal antibody(Enzo Life Sciences ADI-SPA-85-OPE,1:500 for FC).

Validation

Vendor validation information is available online for the following antibodies:

Validation

anti-mouse CD3,BioLegent,Cat#100214
anti-mouse CD49b,BioLegent,Cat#108908
anti-mouse NK1.1,BioLegent,Cat#108713
anti-mouse/human CD11b,BioLegent,Cat#101263
anti-mouse CD45,BioLegent,Cat#103108
anti-mouse CD11c,BioLegent,Cat#117333
anti-mouse F4/80BioLegent,Cat#123149
anti-mouse CD27,BioLegent,Cat#124229
anti-mouse KLRG1,BioLegent,Cat#138410
anti-mouse CD122,BioLegent,Cat#123219
anti-mouse NKp46,BioLegent,Cat#137611
anti-mouse IFN-γ,BioLegent,Cat#505832
anti-mouse CD19,BioLegent,Cat#115507
anti-mouse CD4,BioLegent,Cat#100433
anti-mouse CD49a,BioLegent,Cat#142605
anti-mouse CD127,BioLegent,Cat#135307
anti-mouse CD107a,BioLegent,Cat#121611
anti-mouse CD27,BioLegent,Cat#302824
anti-Eomes,Thermo Fisher Scientific,Cat#53-4875-82
anti-Id2,Thermo Fisher Scientific,Cat#17-9475-82
anti-T-bet,Thermo Fisher Scientific,Cat#12-5825-80
anti-Foxp3,Thermo Fisher Scientific,Cat#17-5773-82
anti-CD8a,Thermo Fisher Scientific,Cat#45-0081-80
anti-human EOMES,Thermo Fisher Scientific,Cat#12-4877-42
anti-human CD3,Thermo Fisher Scientific,Cat#11-0038-80
anti-human CD56,Thermo Fisher Scientific,Cat#17-0567-42
anti-human CD45,Thermo Fisher Scientific,Cat#45-0459-41
PE-conjugated grp95 monoclonal antibody,Enzo Life Sciences,Cat#ADI-SPA-85-OPE
anti-GAPDH,Cell Signaling Technology,Cat#5174
anti-β-actin,Cell Signaling Technology,Cat#3700
anti-phospho-S6,Cell Signaling Technology,Cat#4858
anti-phosphp-Stat5(Tyr694),Cell Signaling Technology,Cat#4322
anti-gp96,Cell Signaling Technology ,Cat#20292
anti-Trim28,Cell Signaling Technology,Cat#4124
anti-His-Tag,Cell Signaling Technology,Cat#12698
anti-α-Tubulin,Cell Signaling Technology,Cat#3873
anti-Calregulin,Cell Signaling Technology,Cat#sc-166837
anti-gp96,Santa Cruz Biotechnology,Cat#sc-393402
anti-Eomes,eBioscience,Cat#14-4875-82
anti-Eomes,Abcam,Cat#Ab216870
anti-Myc-tag,Bioworld,Cat#AP0031M
anti-DDDDK-tag,Bioworld,Cat#AP0007M
anti-GFP-tag,Bioworld,Cat#AP0675M
anti-HA-tag,Bioworld,Cat#AP0005M
Alexa-Fluor 488 Goat Anti-Rabbit lgG,Cell Signaling Technology,Cat#4412
Alexa-Fluor 488 Goat Anti-Mouse lgG,Cell Signaling Technology,Cat#4408
Alexa-Fluor 594 Goat Anti-Rabbit lgG,Cell Signaling Technology,Cat#8889
Alexa-Fluor 594 Goat Anti-Mouse lgG,Cell Signaling Technology,Cat#8890
HRP-Goat Anti-Mouse lgG,Cell Signaling Technology,Cat#7076
HRP-Goat Anti-Rabbit lgG,Cell Signaling Technology,Cat#7074
HRP-Goat Anti-Rat lgG,Cell Signaling Technology,Cat#7077

Eukaryotic cell lines

Policy information about cell lines and Sex and Gender in Research

Cell line source(s)

B16F10 cells from our laboratory were cultured in-house. The HEK293 cell line, NK92 human NK cell lines, MC38 colon cancer cells and LLC lung cancer cells were purchased from the Cell Resource Center, Peking Union Medical College.

Authentication

Its species' origin was confirmed with PCR.The cell line's identity was authenticated with STR profilling(FBI,CODIS).All the resukt can be viewed on the website(http://cellresoure.cn)

Mycoplasma contamination

The cell line was checked free of mycoplasma contamination by PCR and culture.

Commonly misidentified lines
(See ICLAC register)

None of the cell lines used in this study are listed in the datebase of commonly misidentified cell lines maintained by ICLAC.

Palaeontology and Archaeology

Specimen provenance	
Specimen deposition	
Dating methods	

☐ Tick this box to confirm that the raw and calibrated dates are available in the paper or in Supplementary Information.

Ethics oversight	

Note that full information on the approval of the study protocol must also be provided in the manuscript.

Animals and other research organisms

Policy information about studies involving animals; ARRIVE guidelines recommended for reporting animal research, and Sex and Gender in Research

Laboratory animals	Gp96fl/fl mice were provided by Professor Zihai Li (Ohio State University, United States of America). Ncr1-cre mice were purchased from Biocytogen (Beijing, China). Gp96fl/fl mice were crossed with Ncr1-cre mice to generate Ncr1cregp96fl/fl (referred as KO mice). 8 to 12-week-old specific pathogen-free mice were housed at 22±2 ? and 50-60% relative humidity in a specific pathogen-free facility maintained on a 12 hours light/dark cycle at the research animal facility of the Institute of Microbiology, Chinese Academy of Sciences.
Wild animals	No wild animals were used in this study.
Reporting on sex	We used sex-matched mice in this study.
Field-collected samples	The study did not involve samples collected from the field.
Ethics oversight	Animal studies were carried out according to the guidelines set forth by the Institute of Microbiology, Chinese Academy of Sciences of Research Ethics Committee under the approved protocol numbers PZIMCAS2011001.

Note that full information on the approval of the study protocol must also be provided in the manuscript.

Clinical data

Policy information about clinical studies

All manuscripts should comply with the ICMJE guidelines for publication of clinical research and a completed CONSORT checklist must be included with all submissions.

Clinical trial registration	
Study protocol	
Data collection	
Outcomes	

Dual use research of concern

Policy information about dual use research of concern

Hazards

Could the accidental, deliberate or reckless misuse of agents or technologies generated in the work, or the application of information presented in the manuscript, pose a threat to:

No	Yes	
☐	☐	Public health
☐	☐	National security
☐	☐	Crops and/or livestock
☐	☐	Ecosystems
☐	☐	Any other significant area

Experiments of concern

Does the work involve any of these experiments of concern:

No	Yes	
☐	☐	Demonstrate how to render a vaccine ineffective
☐	☐	Confer resistance to therapeutically useful antibiotics or antiviral agents
☐	☐	Enhance the virulence of a pathogen or render a nonpathogen virulent
☐	☐	Increase transmissibility of a pathogen
☐	☐	Alter the host range of a pathogen
☐	☐	Enable evasion of diagnostic/detection modalities
☐	☐	Enable the weaponization of a biological agent or toxin
☐	☐	Any other potentially harmful combination of experiments and agents

Plants

Seed stocks

Novel plant genotypes

Authentication

ChIP-seq

Data deposition

☐ Confirm that both raw and final processed data have been deposited in a public database such as GEO.

☐ Confirm that you have deposited or provided access to graph files (e.g. BED files) for the called peaks.

Data access links
May remain private before publication.

Files in database submission

Genome browser session
(e.g. UCSC)

Methodology

Replicates

Sequencing depth

Antibodies

Peak calling parameters

Data quality

Software

Flow Cytometry

Plots

Confirm that:

☐ The axis labels state the marker and fluorochrome used (e.g. CD4-FITC).

☐ The axis scales are clearly visible. Include numbers along axes only for bottom left plot of group (a 'group' is an analysis of identical markers).

☒ All plots are contour plots with outliers or pseudocolor plots.

☒ A numerical value for number of cells or percentage (with statistics) is provided.

Methodology

Sample preparation	Mice spleen and peripheral lymph nodes were squashed into single-cell suspensions gently through a 70-um cell stainer;Bone marrow cells were flushed out of tibia and femur.After lysis of the red blood cells ,the cell suspensions could be used for indicated experiments.
Instrument	BD Arialll or BD Fortessa
Software	FlowJo was uesd to gate the target populations,and GraphPad Prism was used for further analysis .
Cell population abundance	Freshly isolated splenocytes were stained with anti-CD3(17A2),and anti-NK1.1(PK136)antibodies and NK cells were sorted to>99% purity using a BD FACSArialll.
Gating strategy	For all the experiments involving flow cytometry ,the following gating strategy was applied to eliminate non-specifically stained cells,First,lymphocyte gate was applied on the SSC-A/FSC-A window.Subsequently,sequential singlet/doublet discrimination wsa applied via SSC-W/SSC-H and FSC-W/FSC-H.From the selected singlets,dead cells were discriminated out by gating on live/dead dve negative population.Then the identification of specific cell subsets was detailed in the figure.

☒ Tick this box to confirm that a figure exemplifying the gating strategy is provided in the Supplementary Information.

Magnetic resonance imaging

Experimental design

Design type	
Design specifications	
Behavioral performance measures	

Acquisition

Imaging type(s)	
Field strength	
Sequence & imaging parameters	
Area of acquisition	

Diffusion MRI ☐ Used ☐ Not used

Preprocessing

Preprocessing software	
Normalization	
Normalization template	
Noise and artifact removal	
Volume censoring	

Statistical modeling & inference

Model type and settings

Effect(s) tested

Specify type of analysis: ☐ Whole brain ☐ ROI-based ☐ Both

Statistic type for inference

(See Eklund et al. 2016)

Correction

Models & analysis

n/a	Involved in the study
☐	☐ Functional and/or effective connectivity
☐	☐ Graph analysis
☐	☐ Multivariate modeling or predictive analysis

Functional and/or effective connectivity

Graph analysis

Multivariate modeling and predictive analysis
